# Medical Education and the London University

**Published:** 1908-01-04

**Authors:** T. Gregory Foster

**Affiliations:** Provost. University College, Gower Street, London, W.C.


### Editor's Letter-Box.

### MEDICAL EDUCATION AND THE LONDON UNIVERSITY.

Sir,—I am obliged to you for inserting my letter of December 4 in your issue of December 14. The note that you append to it seems to call for a further letter, and I therefore venture to ask you to afford me the necessary opportunity for replying to that note.

In reference to the scheme for the concentration of Preliminary and Intermediate Medical Studies, you again refer "to the original scheme in the minds of its creators." May I ask who the creators of that scheme were, and in what form the scheme was put forward and what authority it obtained? I have followed the whole movement very closely, and except for the verbal communications of a few individuals, I have never heard of a scheme being put forward and definitely formulated in the shape to which you refer.

With reference to the separation of University College and University College Hospital Medical School, the fact that University College Hospital Medical School is on the opposite side of the same road as University College may for the time being tend to give colour to the view that University College is not a neutral centre for Preliminary and Intermediate Medical Studies. As the organisation of University teaching in London develops, and as the meaning of the Incorporation of University College in the University becomes more fully understood, I am convinced that that difficulty will disappear. Your statement, moreover, overlooks the fact that those Schools that have given up their Preliminary and Intermediate Medical Departments enter their students for those studies at University College, and that the transfer of those students to University College Hospital Medical School is therefore impossible. For example, the St. George's men who come here are entered here by the authorities of St. George's Hospital, who stand *in loco parentium* as far as University College is concerned to those students, all reports and communications with regard to those students being made through the Dean of St. George's Hospital; and similarly in the future with the students who enter here through University College Hospital Medical School or through any other Medical School that desires to send its students to University College for their Preliminary and Intermediate medical studies.

The fact that such of the Fellows, Governors, or Life Governors of University College, London, as desired to become members of the Corporation of University College Hospital were entitled to do so does not prove any connection between the College as now organised and the Medical School. By the Act of Incorporation the Fellows, Governors, and Life Governors of University College, London, ceased to be a body corporate, the College and all its belongings being transferred to the University of London. On the other hand, as the University could not take over the Medical School and Hospital a new corporation was constituted for the government of the Medical School and Hospital. Seeing that many of the Fellows, Governors, and Life Governors of the old Corporation of University College, London, were specially interested in the Medical School, it was surely the right and proper way to secure the continuity of their interest and the continuity of the tradition of that School to enable them to become members of the new Corporation. As the old College Corporation ceased to exist this power does not in any way invalidate the principle of the separation of the College and the Medical School.

You refer to the scheme that is on foot for the purchase of a new athletic ground for the joint use of the clubs of University College and the clubs of University College Hospital Medical School. This scheme was originated entirely by the Students' Union Society before the separation of the College and the Medical School. When the time for the separation became imminent the scheme was modified to suit the new conditions. As the scheme now stands there is no reason why, for example, the Middlesex Hospital Medical School, or for that matter any other School of the University of London that finds the new ground at Perivale convenient for its purpose, should not combine for the acquisition of that ground. The separation between the Medical School and the College has been loyally carried out, as I have said before, but it does not prevent the recognition of the fact that while the College is an integral part of the University of London the Medical School is a School of the University of London. It is, I am sure you will agree, desirable that there should be relations of an intimate character between the University and all its Schools. Nothing could promote that more readily than the acquisition of athletic grounds near together.

The comments that you have made tend only to show that the objects of incorporation have not been fully appreciated. Shortly put the position was this. The University of London was reconstituted and made into a teaching University on paper. The members of the Corporation of University College felt that the intentions of their founders and benefactors could only be fully realised by identifying the College completely with what it is to be hoped will within a reasonable time become a great University organisation. They felt also that the University could only begin to carry out the task imposed upon it if it had control of the resources of the principal University institutions in London; at all events of that part of them that was devoted to what may be described as the *studium generale*. It was in that spirit that the proposal for incorporation was made. It is in a like spirit that the Corporation of King's College have decided to follow the example of University College. The changes that will follow on incorporation ought not to be made rashly and hastily. They should take place slowly. In your article you seem to expect great and evident results within the first year of that important step. To those of us who are at present members of the Senate, and to those of us who are directly concerned with the management of University College, the changes resultant on incorporation that have already taken place are very evident. To the general public they will gradually become evident. I venture, therefore, through your columns, to express the hope that before such criticism as was made in your first article is repeated or made public, fuller knowledge should be obtained and longer time should be given to see results that ought not to be hastened.

What we desire is the creation of a real University in London by the utilisation of all institutions available for that purpose, those institutions being organised in connection with a great scheme. We desire to see the petty jealousies of the past disappear. We desire the promotion of a keen University *esprit de corps*, towards which the intellectual rivalries of the different University Schools, whether of medicine or otherwise, can be a contributing factor.

Yours faithfully,

T. Gregory Foster, Provost.

University College, Gower Street, London, W.C.

December 30, 1907.

[It is, as Dr. Foster must realise, impossible to specify the creators of any scheme of concentration. It is true that no definite scheme has ever been put forward in which it was suggested that University College should abandon the teaching of the Preliminary and Intermediate studies; on the other hand, it is equally a fact that in the communications between the University and the Schools no mention was made of what has evidently been the policy of University College throughout; that they should, whatever happened, retain the teaching of the Preliminary and Intermediate studies. Had this very important fact been mentioned, the University would never have obtained the general expression of opinion in favour of concentration that it did. As was mentioned in our previous note, the incorporation of University College with the University, with the specific mention in the Act of Incorporation that the Preliminary and Intermediate studies should be retained, opened the eyes of the other Medical Schools in London; and the result has been the breakdown of the University scheme of concentration. With reference to the rest of the letter, it may be noted that the facts mentioned in our previous note have not been contradicted; but it is, of course, interesting to have Dr. Gregory Foster's explanation of them, and his view of the benefits that the incorporation of University College is conferring on the other Schools of the University in the Faculty of Medicine. It would also be instructive if he would produce the figures showing how many University College students, who have proceeded to the further study of medicine, have sat for and passed the special Intermediate Science Examination at University College, as compared with the number of students who have passed the Preliminary Scientific (M.B.) Examination, which is general to all students of the University, and for which there is no special internal examination at University College.—Ed. The Hospital.]

